# Impact of soil erosion on agricultural sustainability based on crop water productivity in semi-arid Iran

**DOI:** 10.1038/s41598-025-24353-5

**Published:** 2025-11-18

**Authors:** Elham Rafiei-Sardooi, Fahimeh Mirchooli, Ali Azareh, John J. Clague

**Affiliations:** 1 Department of Ecological Engineering, Faculty of Natural Resources, University of Jiroft, Kerman, 7867161167 Iran; 2 Sari Agricultural Sciences and Natural Resources University, Sari, Iran; 3 Department of Geography, University of Jiroft, Kerman, 7867161167 Iran; 4 Department of Earth Sciences, Institute for Quaternary Research, Simon Fraser University, British Columbia, Burnaby, Canada

**Keywords:** Erosion, Food security, Machine learning, Halil river watershed, Iran, Environmental impact, Sedimentology

## Abstract

Soil erosion is a significant threat to global food production, reducing the productivity of natural ecosystems and agricultural lands. In this study, we examine the loss of crop water productivity in the Halil River agricultural watershed, Iran. Areas within this watershed that are prone to soil erosion were delineated using two machine learning algorithms viz. Support Vector Machines (SVM) and Multivariate Discriminant Analysis (MDA), and 11 geo-environmental factors including elevation, lithology, land use, hydrologic soil group, soil depth, drainage density, soil available water capacity (SAWC), population density, slope (degrees), R factor, and distance to road. Finally, the loss of blue, green, and total crop water productivity of some main cultivated crops (wheat, dates, citrus, and tomatoes) in the study watershed was assessed under pessimistic (20%), optimistic (10%), and normal (15%) scenarios of crop water productivity loss. The results indicate that hydrologic soil group, elevation, and land use are the most important factors for soil erosion susceptibility. In addition, validation results of machine learning algorithms showed that the SVM model (AUC = 94%, TSS = 0.85) outperformed MDA (AUC = 92.3%, TSS = 0.81), and was therefore selected for further analysis. According to the SVM model, 14.3% of the watershed falls within the very high erosion susceptibility class. Agricultural lands are mostly located in areas of moderate to very high erosion risk. Production of wheat, citrus, dates, and tomatoes in moderate, high, and very high areas of erosion susceptibility map are estimated to be 2.4, 5.3, 13.3, and 10.7 million tons, respectively. In the optimistic scenario, total productivity losses per unit of water consumed water by wheat, dates, citrus, and tomatoes are 0.22, 1.24, 2.15, and 2.35 kg m^−3^. and total economic loss will be 14,371, 93,675, 83,247 and 51,893(×10^4^) US$. In the more realistic scenario, these losses are, respectively, 0.32, 1.86, 3.23, and 3.53 kg per unit of consumed water and the economic loss are 21,557, 140,517, 124,872 and 77,841(×10^4^) US$. The total productivity losses per unit of water consumed in the pessimistic scenario are, respectively, 0.43, 2.47, 4.30, and 4.70 kg m^−3^ and total economic loss are 28,743, 187,353, 166,494 and 103,782 (×10^4^) US$. The results underscore the urgent need for site-specific erosion mitigation strategies to safeguard agricultural productivity and water efficiency.

## Introduction

The total human population is predicted to reach 9 billion by about 2050, which are raising concerns about future food security^1^. The UN Food and Agriculture Organization (FAO) estimates that there will be a 44% growth in demand for all cereal crops from 2005 to 2007 to 2050^2^. Food security thus will be one of the most pressing problems faced by humanity over the next several decades and is of particular concern in arid and semi-arid countries, which are at risk from climate change^3^. Increasing water productivity in countries with limited water supplies is a key priority for governments aiming to boost crop production to satisfy future food requirments^4^. In this context, soil provides a wide range of ecosystem services that are critically important in meeting basic human food needs^5^. However, globally soils are subject to unprecedented degradation and erosion^6–8^. In particular, soil erosion is reducing our collective ability to increase food production to the level required to feed 9 billion people by mid-century. FAO has identified soil erosion as one of the top ten risks to food security^9^.

Soil erosion has additional adverse on-site and off-site consequences, including river siltation, flooding, soil nutrient loss, water pollution, and reduced agricultural productivity^5^. To control these problems, erosion-prone areas and the factors responsible for soil erosion must be identified, and agricultural systems in erosion-prone areas must be sustainably managed.

Iran ranks highest among Asian countries suffering from soil erosion, with approximately 94% of its agricultural land experiencing soil degradation^10^. Insufficient rainfall, low soil organic matter, and overall poor soil quality compound the challenge of maintaining much less increasing agricultural production. Soil erosion not only decreases food production, but also exacerbates desertification and poverty in rural areas of Iran^10^.

In recent years, a variety of equations and methods have been developed to identify and manage erosion-susceptible agricultural areas. Notable examples include the Universal Soil Loss Equation (USLE)^11^, Revised Universal Soil Loss Equation (RUSLE)^12^, Water Erosion Prediction Project Model (WEPP)^13^. Recently, machine learning (ML) algorithms have been used to address the multivariate and complex nature of soil erosion^14–17^ and improve the accuracy of soil erosion maps^18^. In addition, models such as weights of evidence^19^, the generalized linear model^9^, and artificial neural network (ANN)^20,21^, have been successfully applied in soil erosion research. For example, in a study, analytical neural network and geographically weighted regression were used to extract areas vulnerable to soil erosion and identify the most suitable model for soil erosion susceptibility in subtropical environment^20^. In other study, Multi-layer perception approach (MLPC) and its ensembles (MLPC-Bagging, gully erosion^8^. Recently, researchers have combined empirical models with ML algorithms to identify soil erosion hotspots and their causes. For example, in a study, RUSLE was integrated with generalized linear model (GLM), classification tree analysis (CTA), artificial neural network (ANN) and random forest (RF) to improve the identification of regions with soil erosion in the Talar watershed, Iran^9^. Despite these advances, few studies have linked erosion susceptibility mapping to quantitative assessments of agronomic performance.

In general terms, soil erosion is evaluated by measuring losses of soil material due to water, wind, and human modification of the land surface. These losses are can be readily observed and measured. In contrast, intrinsic impacts on chemical properties, soil nutrient content, and biological activity are typically difficult to identify and measure, even though they are equally or even more important for soil productivity and ecosystem function than soil loss itself. All forms of soil degradation markedly affect water availability in both rainfed and irrigated agricultural systems by altering runoff and soil water retention, and by impeding plant’ access to soil water^22^. Crop water productivity (CWP) is an appropriate concept for studying and discussing the impact of soil erosion on irrigated and rainfed crops. It is the ratio of crop yield to the amount of water consumed in the production process. However, there have been numerous studies that have investigated CWP^7,23,24^, little work has been done in Iran and world on the impact of soil erosion on crops and food security based on CWP concept.

To be actionable for food security and water management, erosion assessments should not only identify hotspots but also quantify how erosion alters crop yields and water productivity under different scenarios. We have identified soil erosion-prone areas in the Halil River watershed, Iran, where is critical to the economy of the area. It is an important agricultural area, especially for wheat cultivation. The objectives of our study are threefold: (1) to produce an accurate soil erosion susceptibility map of the watershed using machine learning algorithms; (2) to select the most important factors affecting soil erosion in the watershed; and (3) to calculate losses of crop water productivity under three different scenarios of erosion based on the CWP concept.

## Study area

**Fig. 1:**
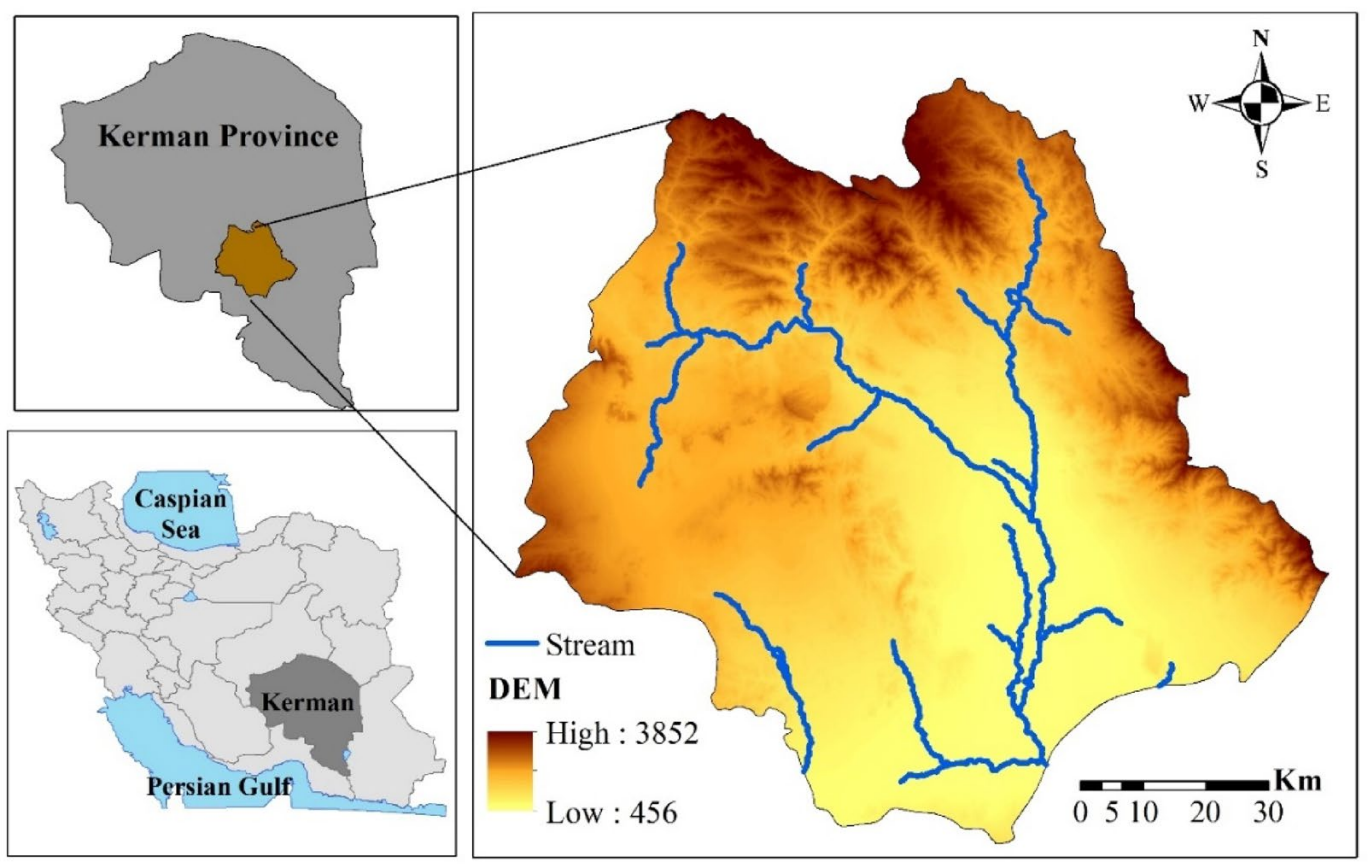
Location of the study area in Kerman Province, Iran (The data used to create this figure were derived from Iran National Cartographic Center (https://ncc.gov.ir), and the figure was created using ArcGIS software (version 10.8, https://www.esri.com).

The Halil River watershed (9204 km^2^) is located in Kerman Province in southeast Iran. It extends from 28°05´N to 29°15´N and 56°55´E to 58°15´E (Fig. 1) and gradually rises in elevation from 498 m to 3868 m asl (above sea level) from south to north. The watershed serves as a tributary to the Jazmourian wetland, which provides surface water that is essential for local agriculture.

The climate in the region is arid to semi-arid^25^. Average annual precipitation ranges from 155 mm to to 4254 mm, and mean annual temperature ranges of 16.7 to 25.8 ^◦^C.

The Halil River watershed is home to about 390,000 persons distributed across 24 local communities^26^. The population depends heavily on agriculture, with the watershed playing a crucial role in crop production in Iran. Additionally, the study area supports a diverse variety of vegetation types, including irrigated orchards, annually irrigated crops, and natural vegetation. The irrigation of crops such as wheat, barley, corn, watermelon, cucumber, potato, onion, and tomato represents the most water-demanding form of land use. Orchards are primarily composed of citrus trees, while the natural vegetation, which covers a significant portion of the study area, is mainly used for livestock grazing^27^.

## Methods

**Fig. 2:**
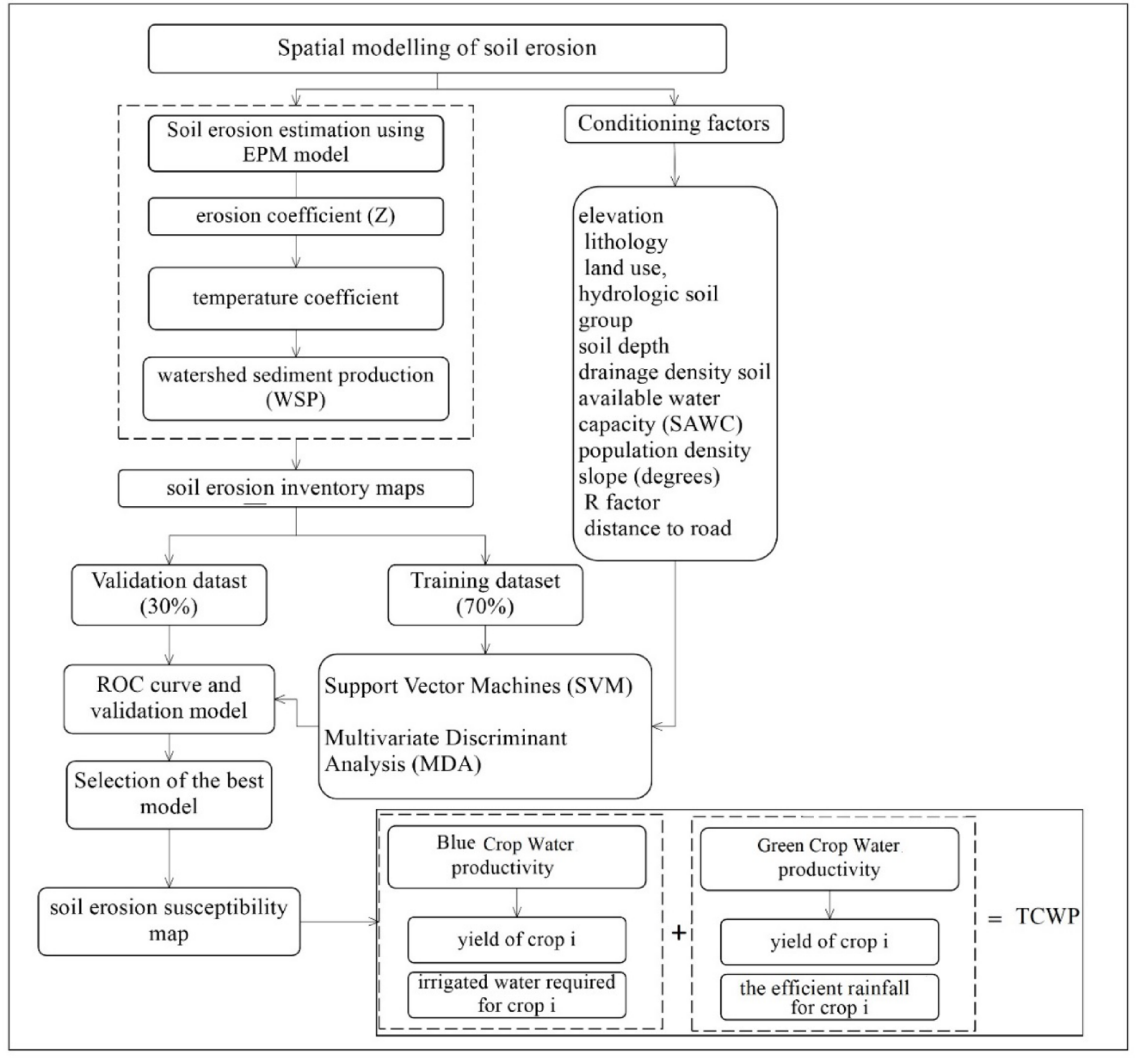
Flowchart of the current study.

This study consists of three main steps: (1) assessment of the soil erosion using EPM model in the study area. (2) Data preparation and spatial modeling. (3) Assessment of crop water productivity. The respective flowchart is presented in Fig. 2.

### Soil erosion Estimation

The erosion potential method (EPM) was firstly introduced by Gavrilović^28^.This model can be used to estimate the severity of erosion and to prepare soil erosion inventory map in watersheds^29^.

The EPM model was selected primarily due to its lower sensitivity to topographic parameters^30^. Unlike RUSLE, which explicitly incorporates a slope-length (LS) factor to account for erosion dynamics, the EPM model does not rely on this component. While the LS factor makes RUSLE effective in areas with relatively gentle terrain, it also introduces bias when applied to steep slopes, where erosion tends to be overestimated^8^. Given the presence of steep terrain within the study area, the reduced topographic sensitivity of the EPM model makes it more suitable and reliable for accurate erosion assessment.

The soil, surface geology, land use, topographic features and climatic factors (including mean annual rainfall, and mean annual temperature) are main variables which can be applied in EPM model^31–33^.Although the method can provide average annual soil erosion rates, it is not suitable for assessing erosion on a specific event basis^34^.The method estimates the coefficient of erosion intensity (Z) in a watershed using the following Eq. 2^35^:

1 $$\:Z=Y\times\:X\times\:\psi\:\times\:\sqrt{I}$$

where Y is the coefficient of soil erodibility, X is land use coefficient, ψ is the coefficient of the type of erosion processes, and I is the average watershed slope gradient. According to Gavrilovic^28^, Y, X, and ψ can be estimated using descriptive and numerical data. The required data are summarized in Table 1, based on Dragičević et al^36^. and Baharvand and Pardhan^29^.

**Table 1:** The contributing factors in EMP model to estimate the soil erosion.

Parameters and classes	Values
Land use coefficient (X)	
Forest	0.2
Pasture land	0.4
Farmland	0.6
Bare land	0.8
Soil erodibility (Y)	
Dolomite	0.4
Limestone	0.6
Sandstone	0.8
Conglomerate	1.0
Shale and marl	1.3
Loess deposits	1.8
Type of erosion ($\:\psi\:$)	
Hard rocks	0.2
Karstic erosion	0.5
Gully and surface erosion	0.8

The erosion potential method estimates average annual gross soil erosion (Wsp) (m3/km^−2^.yr^−1^) using Eq. (2)^36,37^:

2 $$\:Wsp\:=\:T\times\:H\times\:\pi\:\times\:\sqrt[3]{Z}\times\:A$$

where H is mean annual amount of precipitation (mm/year), π is equal to 3.14, Z is the coefficient of erosion intensity calculated from Eq. (1), A is the area of the study, and T is a temperature coefficient quantified from the mean annual temperature (*t)* according to Eq. (3):

3 $$\:T=\sqrt{\frac{t}{10}+0.1}$$

**Fig. 3:**
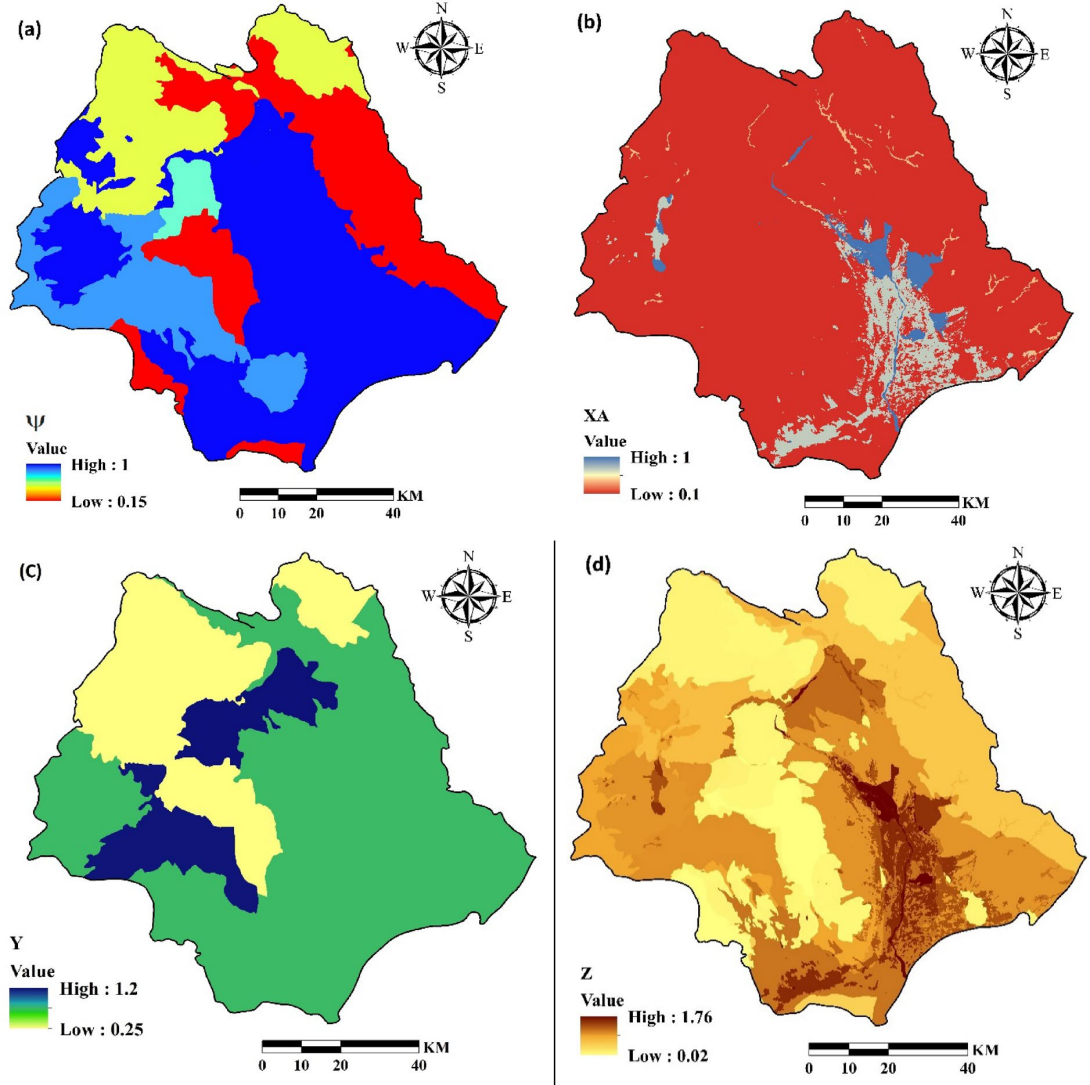

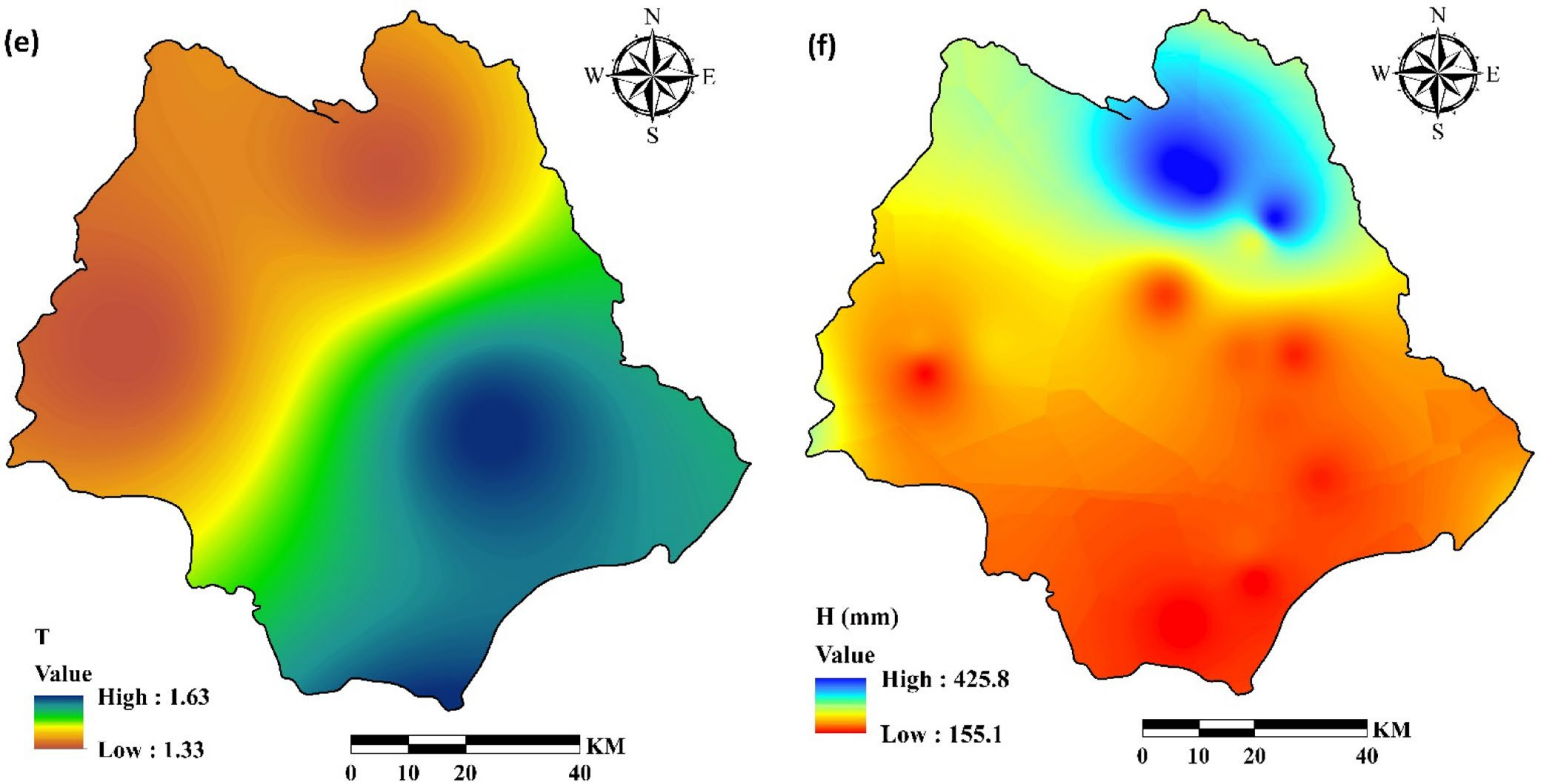
Estimates of the EPM parameters in the study watershed, including (**a**) erosion coefficient (ψ), (**b**) land use coefficient (X), (**c**) soil erodibility coefficient (Y), (**d**) erosion coefficient (Z), (**e**) temperature coefficient and (**f**) mean annual rainfall (H) (The data used including ψ, X and Y were obtained from Iran National Cartographic Center (https://ncc.gov.ir), T and H were obtained from Iran Water Resources Management (WRM) company (https://data.wrm.ir), and the figures were generated using ArcGIS software (version 10.8, https://www.esri.com).

Figure 3 shows the EPM parameters including erosion coefficient (ψ), land use coefficient (X), soil erodibility coefficient (Y), erosion coefficient (Z), temperature coefficient and mean annual rainfall (H). All variables were adjusted to a consistent spatial resolution of 30 m.

The value of Wsp provides an estimate of the total amount of soil eroded in a watershed due to several erosion processes (e.g., rill, sheet, gully erosion) and smaller soil slumps^32^.

Where, P is circumference of the watershed, L is watershed length (Km), D is height difference in the watershed area (Km). After calculation of Ru value, the special sediment rate is estimated by equations Eqs. 5, 6 and 7:

4 $$Gsp=Wsp \times Ru$$

5 $$Ru=\frac{{4 \times {{\left( {P \times D} \right)}^{0.5}}}}{{L+10}}$$

6 $$\text{GS=GSP.F}$$

Where Gsp is special sediment rate, Ru is sedimentation coefficient, GS is total sediment rate (m^3^/yr), F is total watershed area (km^2^)^28^.

To assess the performance of the EPM model, the average estimated sediment was compared with the average observed sediment at the Kahnak –e Sheibani station at the basin outlet.

After preparing soil erosion map, the EPM map was categorized by erosion rates and areas > 16 ton/ha/year were considered as regions with severe erosion^9^. These regions were extracted, and 300 points were chosen to be as occurrence points of soil erosion for the ML models. To build our ML model, 70% of the total dataset was used for calibration and training, while the remaining 30% were used for model validation^38,39^. The average soil erosion rate in Iran is 15–20 tons ha^−1^year^−1^. This is 3 to 4 times higher than the global average due to climatic, topographic, and anthropogenic conditions^40,41^.

### Dataset Preparation for Spatial soil erosion modeling

Meteorological and topographical factors, as well as vegetation and soil characteristics influence soil erosion. Eleven geo-environmental factors including elevation, lithology, land use, hydrologic soil group, soil depth, drainage density, soil available water capacity (SAWC), population density, slope, rainfall erosivity (R factor), and distance to road were used in this study to categorize and map soil erosion. These factors were chosen based on previous studies^9,35,42,43^, data availability, and the authors’ experience^44^.

**Fig. 4:**
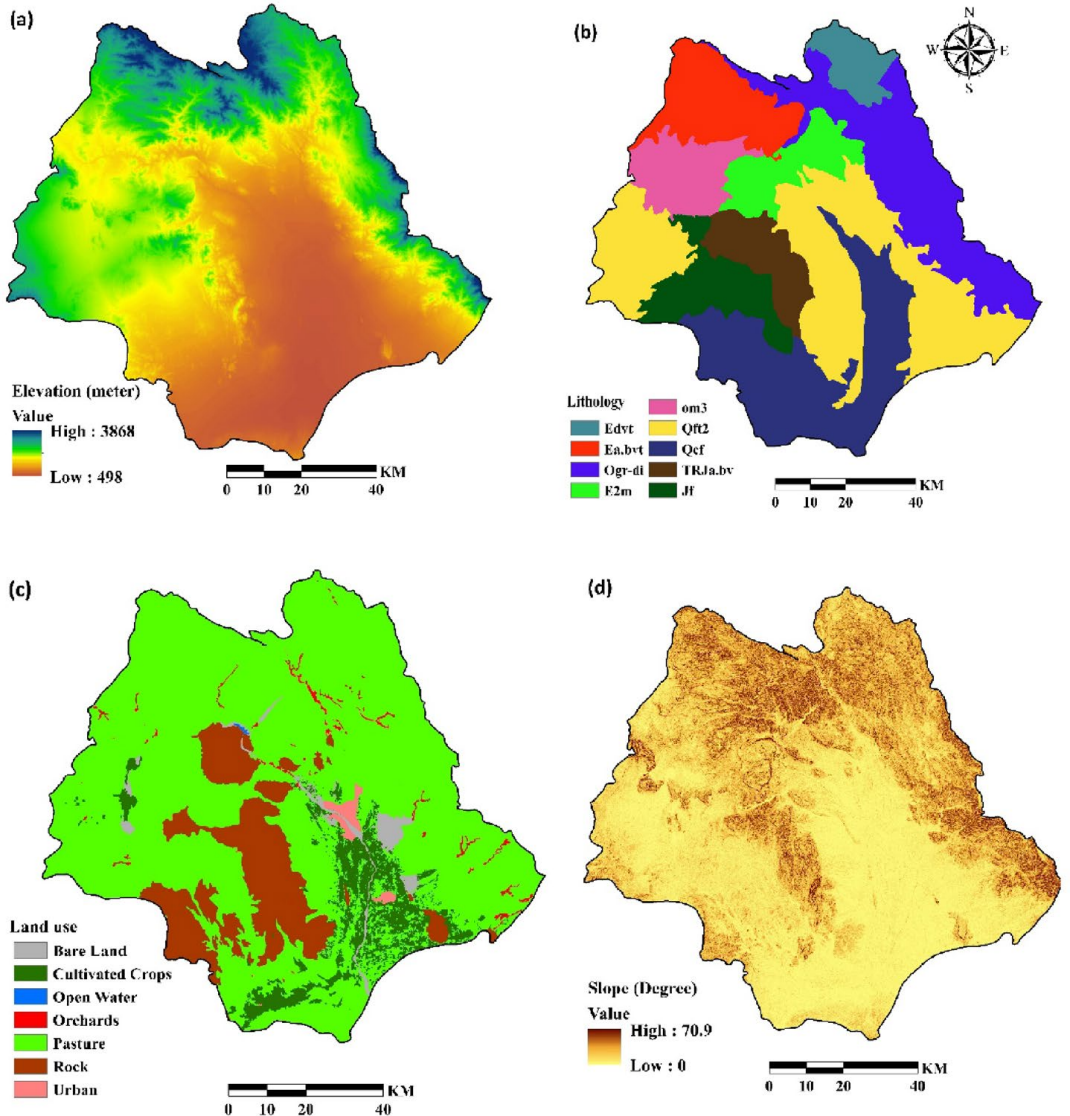

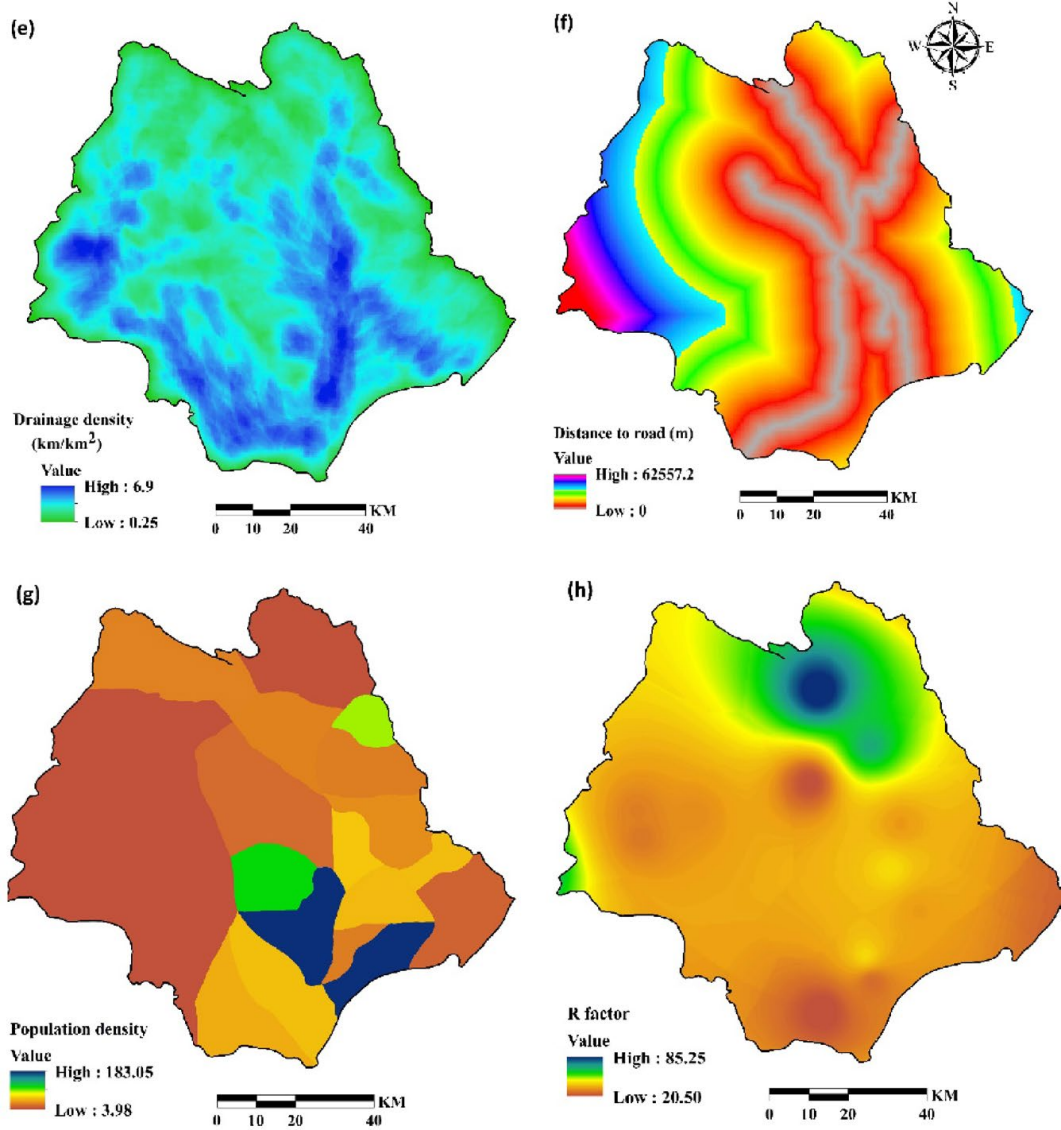

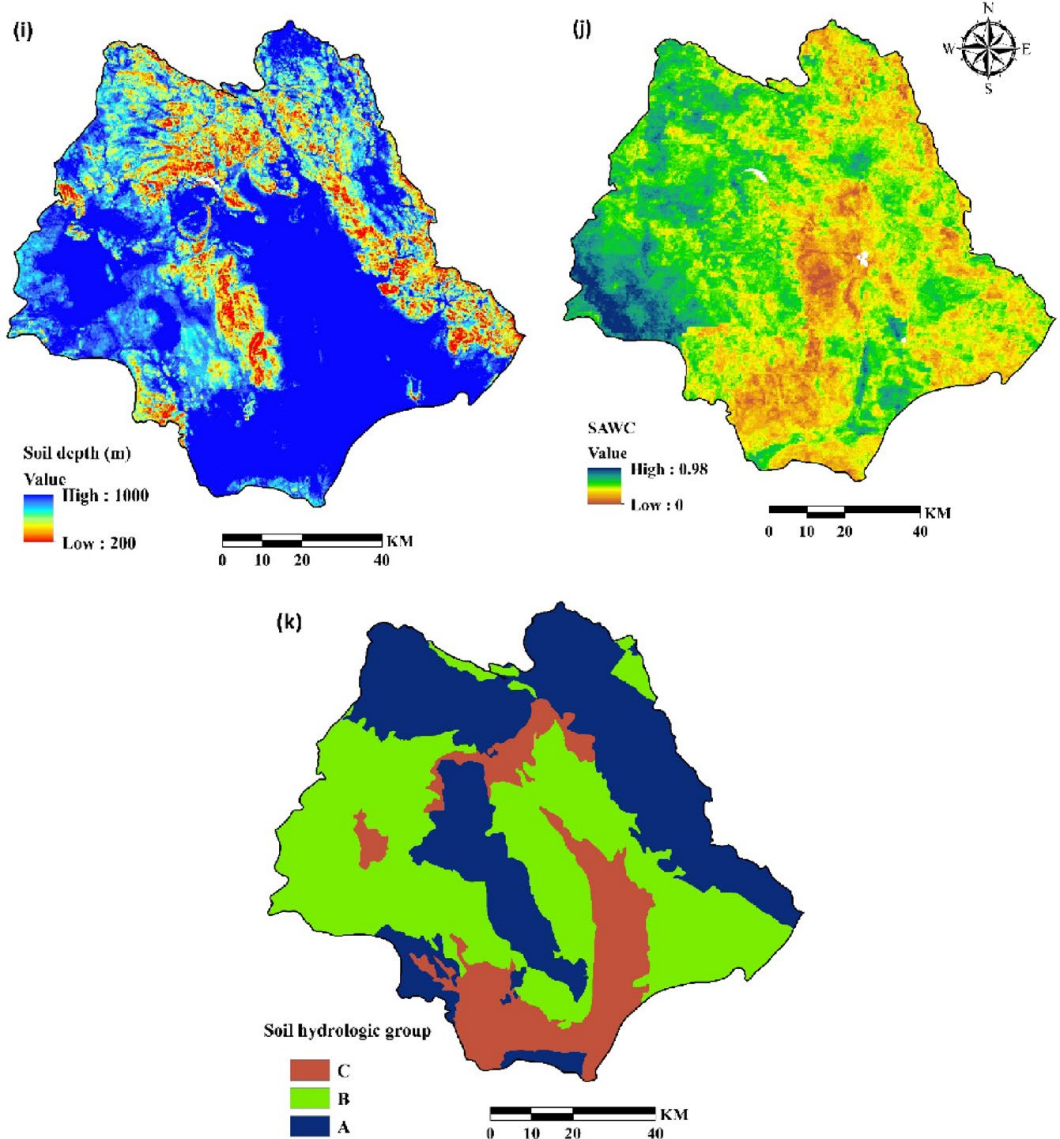
Conditioning factors for soil erosion susceptibility analysis: (**a**) elevation (**b**) lithology (**c**) land use (**d**) slope (**e**) drainage density (**f**) distance to road (**g**) population density (**h**) R factor, (**i**) soil depth, (**j**) SAWC and (**k**) hydrologic soil group (The data used to create the figures (**a**), (**b**), (**c**), (**d**), (**e**) and (**f**) were obtained from Iran National Cartographic Center (https://ncc.gov.ir), the data for the figure (**g**) were obtained from Statistical Centre of Iran (https://amar.org.ir), the data for the figure (**h**) were obtained from Iran Water Resources Management (WRM) company (https://data.wrm.ir), the data for figures (**i**), (**j**) and (**k**) were obtained from field study, finally the figures were generated using ArcGIS software (version 10.8, https://www.esri.com).

Elevation (Fig. 4a) was derived from the DEM and categorized in ArcGIS 10.8. Lithology (Fig. 4b; Table 2) was considered by digitizing geological maps at 1:50,000 scale.

Land use significantly impacts soil erosion. Conversion of natural landscapes to agriculture or urban areas often leads to increased erosion due to factors like deforestation, soil disturbance, and reduced vegetation cover^45,46^.

A land-use map of the Halil River watershed was created (Fig. 4c) from a Landsat 8/Operational Land Imager (OLI) image using a supervised classification algorithm in the ENVI 5.3.

**Table 2:** Lithology classes in the study area.

Code	Lithology	Geological age
TRJa.bv	Andesitic to basaltic volcanic	Early-Middle.Triassic
Jf	Flysch turbidities, sandstone, shale, conglomerate, volcanic rocks and limestone	Jurassic
om3	Pelagic limestone, radiolarian chert and shale in association with basalt	Late.Cretaceous
E2m	Pale red marl, gypsiferous marl and limestone	Eocene
Edvt	Rhyolitic to rhyodacitic volcanic tuff	Eocene
Ea.bvt	Andesitic to basaltic volcanic tuff	Eocene
Ogr-di	Granite to diorite	Oligocene
Qft2	Low level piedmont fan and valley terrace deposits	Quaternary
Qcf	Clay flat	Quaternary
Qft2	Low level piedment fan and valley terrace deposits	Quaternary

Seven land-use types were identified: bare land, cultivated crops, open water, orchards, pasture, rock, and urban. Slopes, ranging from 0 to 72^o^ (Fig. 4d) were derived from a digital elevation model (DEM) of the watershed (Fig. 4a). Drainage density map was obtained from rivers map of the study area (Fig. 4e) using line density command in ArcGIS 10.8. Areas near drainage divides are at high risk of erosion^42,47,48^. Areas near roads are typically susceptible to soil erosion^49,50^, thus a distance-to-road layer (Fig. 4f) was extracted from a 1: 25,000-scale topographic map in ArcGIS 10.8 using the Euclidean distance method. Population density (Fig. 4g) was derived from population statistics provided by Iran Statistical Center Organization^26^. R factor is rainfall erosivity in revised universal soil loss equation (RUSLE) and is a significant aspect in soil erosion modeling^51,52^. It was computed from rainfall data obtained from seven meteorological stations using Renard and Freimund’s^12^ method and then interpolated using the Inverse Distance Weighted (IDW) method in ArcGIS (Fig. 4h). Soil properties, including soil depth (Fig. 4i), soil available water capacity (SAWC) (Fig. 4j) and soil hydrological group (Fig. 4k) were obtained from 79 soil samples extracted in a field. Table 3 show the variable types and scale of all mentioned data used for the study. Finally, all variables were adjusted to a consistent spatial resolution of 30 m.

**Table 3:** Data used for the study and analysis.

Factors	Variable type	Scale	Data source
Elevation (m asl)	Continuous	30*30 m	DEM (ASTER satellite) (https://earthexplorer.usgs.gov)
Lithology	Categorical	1:50,000	Geological Survey and Mineral Exploration of Iran (https://gsi.ir/)
Land use	Categorical	1:50,000	Iran National Cartographic Center (https://ncc.gov.ir/)
Hydrologic soil group	Categorical	1:50,000	Ministry of Agriculture Jihad (https://maj.ir/)
Soil depth (m)	Continuous	30*30 m	Ministry of Agriculture Jihad (https://maj.ir/)
Drainage density (km/km^2^)	Continuous	30*30 m	Geological Survey and Mineral Exploration of Iran (https://gsi.ir/)
SAWC	Continuous	30*30 m	Ministry of Agriculture Jihad (https://maj.ir/)
Population density (person/area)	Continuous	30*30 m	Statistical Centre of Iran (https://amar.org.ir/)
Slope (degrees)	Continuous	30*30 m	DEM (ASTER satellite) (https://earthexplorer.usgs.gov)
R factor	Continuous	30*30 m	Iran Water Resources Management Company (https://www.wrm.ir/)
Distance to road (m)	Continuous	30*30 m	Iran National Cartographic Center (https://ncc.gov.ir/)

### Multi-collinearity analysis

Multi-collinearity analysis consistently provides accurate insights for assessing the linear relationships among various geo-environmental factors within a machine learning model. This statistical technique identifies pairs of variables that exhibit a high correlation in multiple regression analyses^44^. Therefore, examining multi-collinearity is crucial for enhancing model performance by eliminating highly correlated factors and reducing bias. Researchers worldwide have applied multi-collinearity analysis across diverse fields, including landslide^53^, gully erosion^44^, flood studies^54^. Multi-collinearity can be evaluated using the variance inflation factor (VIF) and tolerance (TOL) metrics. Typically, a TOL value below 0.10 or 0.20, coupled with a VIF value exceeding 5 or 10, indicates significant multi-collinearity among the variables^55^.

### Application of machine learning models

#### Support vector machines (SVM)

**Fig. 5:**
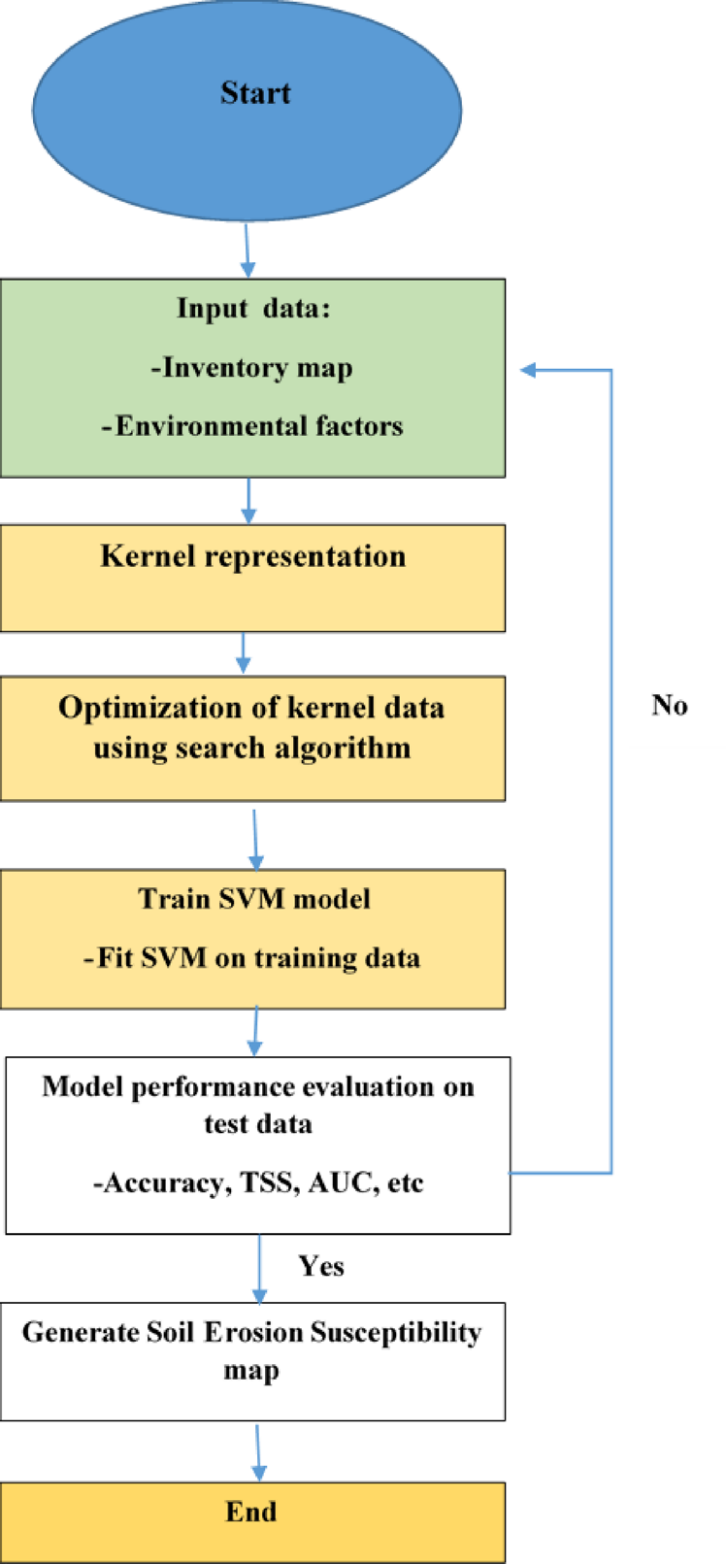
The process flowchart of SVM.

This method is a favored supervised machine-learning algorithm for addressing regression and classification problems^56^. It is designed for binary classification models^54^ and relies on the theory of statistical learning^57^ and the concept of structural risk minimization. It can be applied simultaneously for both regression and classification tasks. The algorithm was executed using R software version 3.5.3 along with the species distribution modeling (SDM) package^58^. Figure 5 shows the process flowchart of SVM.

#### Multivariate discriminant analysis (MDA)

**Fig. 6:**
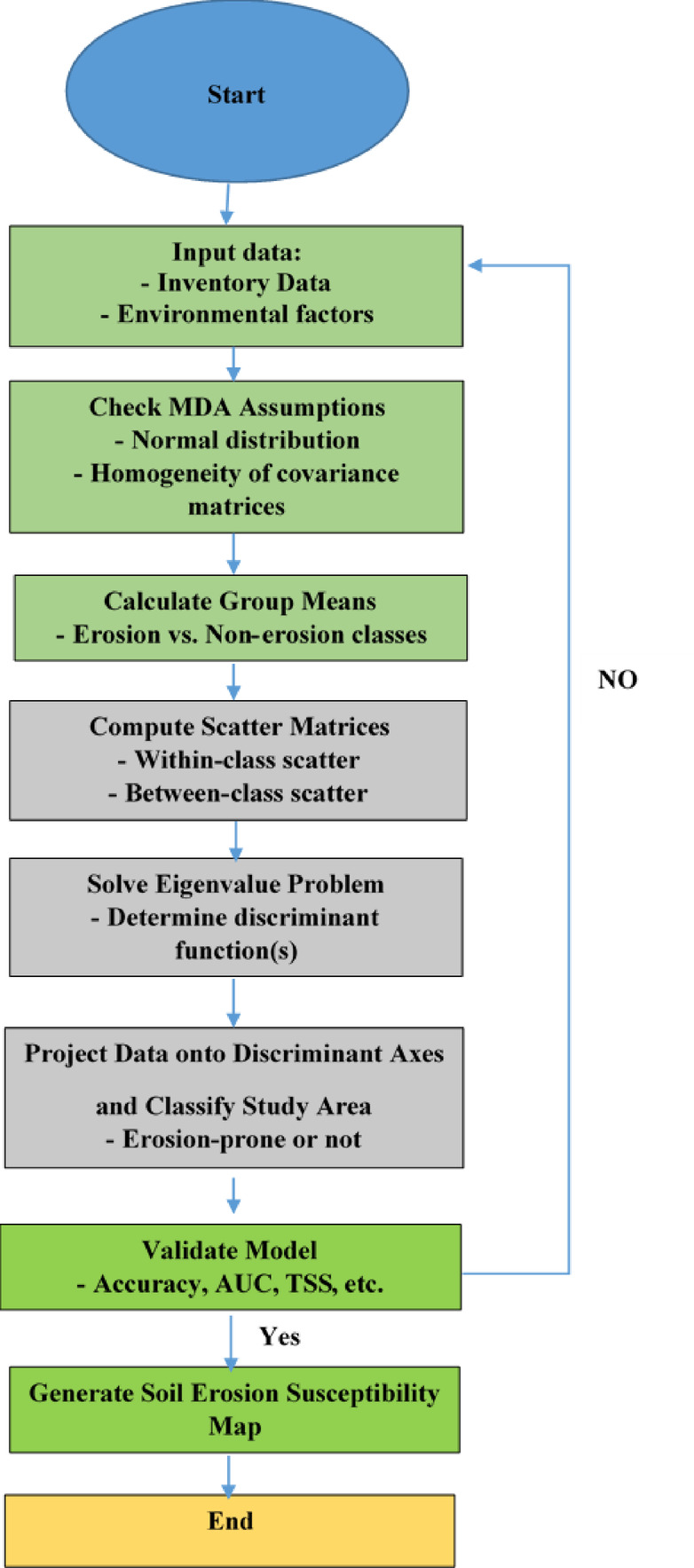
The process flowchart of MDA.

Multiple Discriminant Analysis (MDA) is a statistical method that predicts an independent variable using a set of dependent variables. It combines performance features found in more complex neural network models^59^. MDA generates an equation derived from a linear combination of independent variables^25^. Discriminant coefficients, representing the weight of each independent variable, are adjusted based on the interrelationships among all variables:

7 $$\:{d}_{i}={W}_{i1}{X}_{1}+{W}_{i2}{X}_{2}+\dots\:+{W}_{in}{X}_{n}$$

where *d*_*i*_ is the discriminant score, *X*_*i*_ is the discriminant variable, and *W*_*ij*_ is the discriminant coefficient. The algorithm was executed using R software version 3.5.3 along with the species distribution modeling (SDM) package^58^. Figure 6. shows the process flowchart of MDA.

### Evaluation of accuracy

To assess the performance of the ML models, we used precision, the popular recall (sensitivity), AUC, TSS, F_Score, Overall Accuracy metrics that are calculated using the following Eq. (7) to (12):

8 $$\Pr ecision=\frac{{TP}}{{TP+FP}}$$

9 $$\operatorname{Re} call/Sensitivity=\frac{{TP}}{{TP+FN}}$$

10 $$Specificity=\frac{{TN}}{{FP+TN}}$$

11 $$1 - Specificity=\frac{{FP}}{{FP+TN}}$$

12 $$\text{TSS=Sensitivity+Specificity-1}$$

13 $$F\mathop {}\limits_{ - } Score=2 \times \frac{{\Pr ecision \times \operatorname{Re} call}}{{\Pr ecision+\operatorname{Re} call}}$$

14 $$Overall\mathop {Accuracy=\frac{{TP+TN}}{{TP+TN+FP+FN}}}\limits^{{}}$$

where “TP is true positive, FN is false negative, FP is false positive, sensitivity (true positive rate) is the portion of the positive samples that are properly classified, and 1-specificity (false positive rate) is the portion of the positive samples that are incorrectly classified”^17^.

Recall (sensitivity) represents the proportion of all relevant instances that are correctly identified. Thus, both precision and recall are metrics that evaluate relevance^60^.

The True Skill Statistic (TSS), was firstly proposed by Allouche et al.^35^ is another metric used to evaluate classification accuracy. TSS statistics are based on the ratio of true prediction which includes both true positives and true negatives^61^, with values changing from − 1 to + 1. A value of + 1 is indicative of high classification efficiency, whereas anything lower than 0 is worse than random^62^.

The receiver operating characteristic (ROC) curve has proven to be useful for evaluating the accuracy of hazard susceptibility maps created using machine learning algorithms^63,64^. In this study we assessed the ability of the applied models to accurately map soil erosion using the area under the ROC curve (AUC). In general, AUC values range from 0.5 to 1.0, with a lower value indicating lesser accuracy and higher values signifying stronger models.

Accuracy can be misleading when dealing with imbalanced datasets. For instance, if a dataset contains 95 positive and 5 negative cases, predicting all cases as negative would still result in a 0.95 accuracy score^41^. In contrast, the F1-score, which is the harmonic mean of precision and recall, tends to approximate their average when the two values are similar and generally represents their harmonic mean^65^. Overall accuracy (OA) reflects the likelihood that a test correctly classifies an individual and is calculated as the weighted average of sensitivity and specificity^66^.

### Crop water productivity (CWP)

The impact of soil erosion on agricultural productivity was assessed by extracting agriculture areas from land-use maps and analyzing agricultural statistics. Assessing agricultural damage caused by soil erosion required consideration of scenarios of CWP losses for different cultivated crops grown in areas of different erosion vulnerability.

Based on Iran’s annual agricultural organization report^2^, the main agricultural crops in the Halil River watershed are wheat, dates, citrus, and tomatoes. These crops cover total areas of, respectively, 21.6%, 19.5%, 18.7%, and 10.5% of agricultural and orchard lands in the watershed.

CWP is expressed as the amount of crop produced per unit of water^22^. There are two categories of CWP: blue water drawn from surface or groundwater sources, and green water derived from rainwater^61,67^. Blue crop water productivity was computed using Eq. 13:

15 $$\:BCWP=\frac{{Y}_{\:i}}{{IWR}_{i}}$$

where BCWP is blue crop water productivity, Y_i_ is the yield of crop *i*, and IWR_i_ is irrigated water required for crop *i.*

Green crop water productivity was calculated using Eq. (14):

16 $$\:GCWP=\frac{{Y}_{\:i}}{{Pe}_{i}}$$

where GCWP is green crop water productivity, Y_i_ is the yield of crop *i*, and Pe_i_ is the efficient rainfall for crop *i*.

Finally, total crop water productivity (TCWP) was determined using GCWP and BCWP.

17 $$\:TCWP=GCWP+BCWP$$

In this study, a scenario-based approach has been used to calculate the impacts of soil erosion on CWP. Specifically, we chose three scenarios (optimistic, normal, and pessimistic), in which 10%, 15%, and 20% of CWP were lost due to soil erosion.

Also, economic loss of the main crops production under three scenarios (optimistic, normal, and pessimistic) were estimated. So that, it firstly assumed that 10%, 15%, and 20% of crop production were lost due to soil erosion. Then price of each crop multiplied by its production (tons) and finally, it is compared to initial price.

### Code availability

No custom code was generated for this study. All analyses were conducted using the publicly available ‘sdm’ R package developed by Naimi & Araujo^58^. The package can be accessed and downloaded from the Comprehensive R Archive Network (CRAN) at [DOI: 10.1111/ecog.01881].

## Results

### Soil erosion estimation of the EPM

**Fig. 7:**
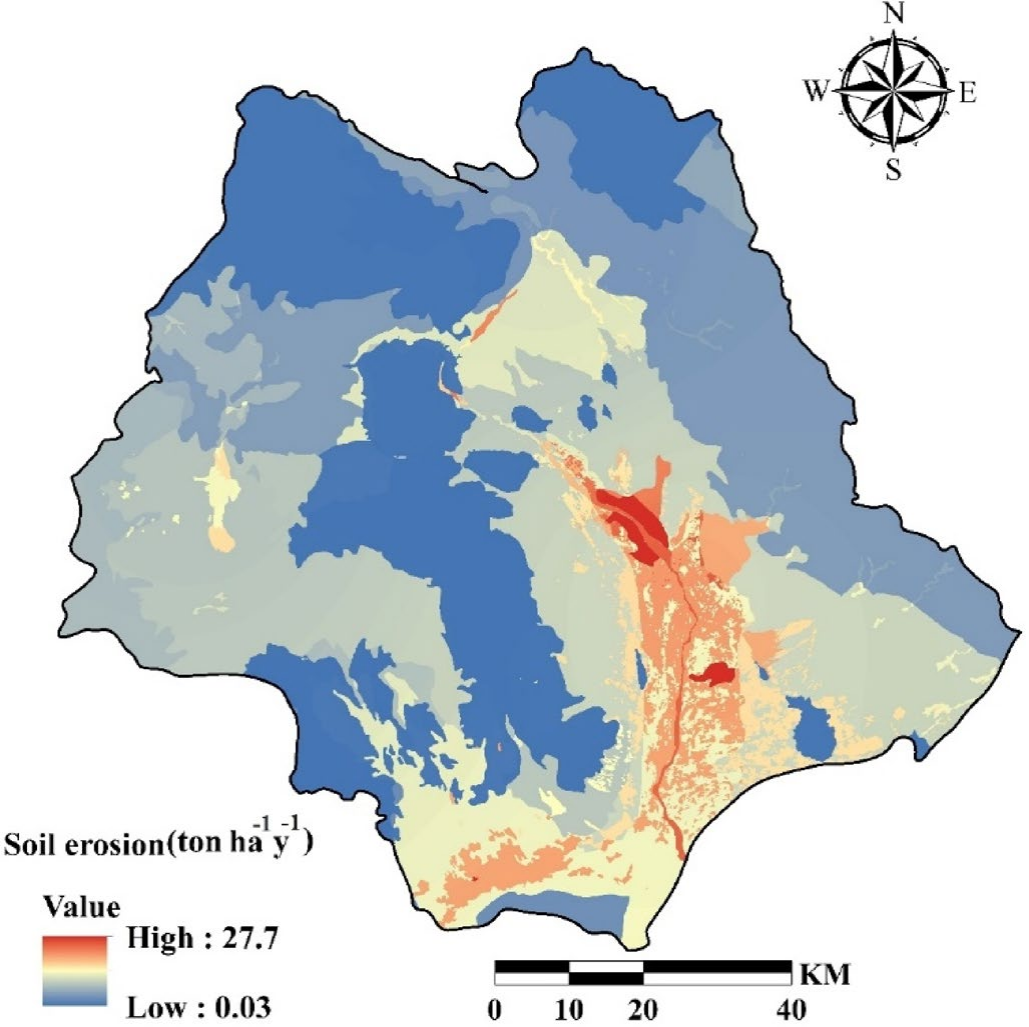
The average annual gross soil erosion obtained from EPM.

Estimates of erosion parameters and coefficients for the Halil River watershed, including.

erosion coefficient of watershed (ψ), land use coefficient (X), the coefficient of soil erodibility (Y), the coefficient of erosion intensity (Z), temperature coefficient (T), mean annual rainfall (H) are summarized in Fig. 3. The average annual gross soil erosion (W) has been shown in Fig. 7. The soil erosion map was prepared with resolution 30 m.

Soil erosion ranges from 0.03 to 27.7 ton ha^−1^year^−1^ and the highest values were distributed in the southern and central parts of the watershed. In addition, the lowest values of the erosion were observed in the northern and eastern of the watershed. For further analysis, erosion-prone locations with the values more than 16 ton ha^−1^ year^−1^ were extracted as severe erosion locations^9^ for model training.

The average soil erosion rate in the study area was estimated at 7.1 t ha^−1^ y^−1^ using EPM and the estimated sediment was 2.8 t ha^−1^ y^−1^ that the Ru was 0.4. The average observed sediment at the Kahnak –e Sheibani station at the basin outlet was 3.4 t ha^−1^ y^−1^. In other words, the observed sediment content was about 21.4% higher than the estimated sediment. Therefore, there was no significant difference between observed and estimated sediment and the EPM model had good performance.

### Multi-collinearity test

Multicollinearity, a major concern in classification and regression analysis, refers to the situation where multiple independent variables within a model are correlated with each other. In the present study, we utilized tolerance (TOL) criteria and the variance inflation factor (VIF) to evaluate collinearity among the conditioning variables. TOL values below 0.1 and VIF values exceeding 10 suggest the presence of multi-collinearity among predictor variables^55^. Table 4 displays the VIF and TOL values for soil erosion predictors in this study, indicates that there is no multicollinearity present among the predictor variables.

**Table 4:** The multi-collinearity analysis among variables.

Variables	Tolerance	VIF
Elevation	0.739	1.34
Lithology	0.645	2.79
Land use	0.602	2.68
Hydrologic soil group	0.821	1.76
Soil depth	0.617	2.65
Drainage density	0.512	2.4
SAWC	0.358	3.27
Population density	0.466	2.65
Slope degree	0.731	1.29
R factor	0.387	4.64
Distance to road	0.784	2.65

### The most important conditioning factors

**Fig. 8:**
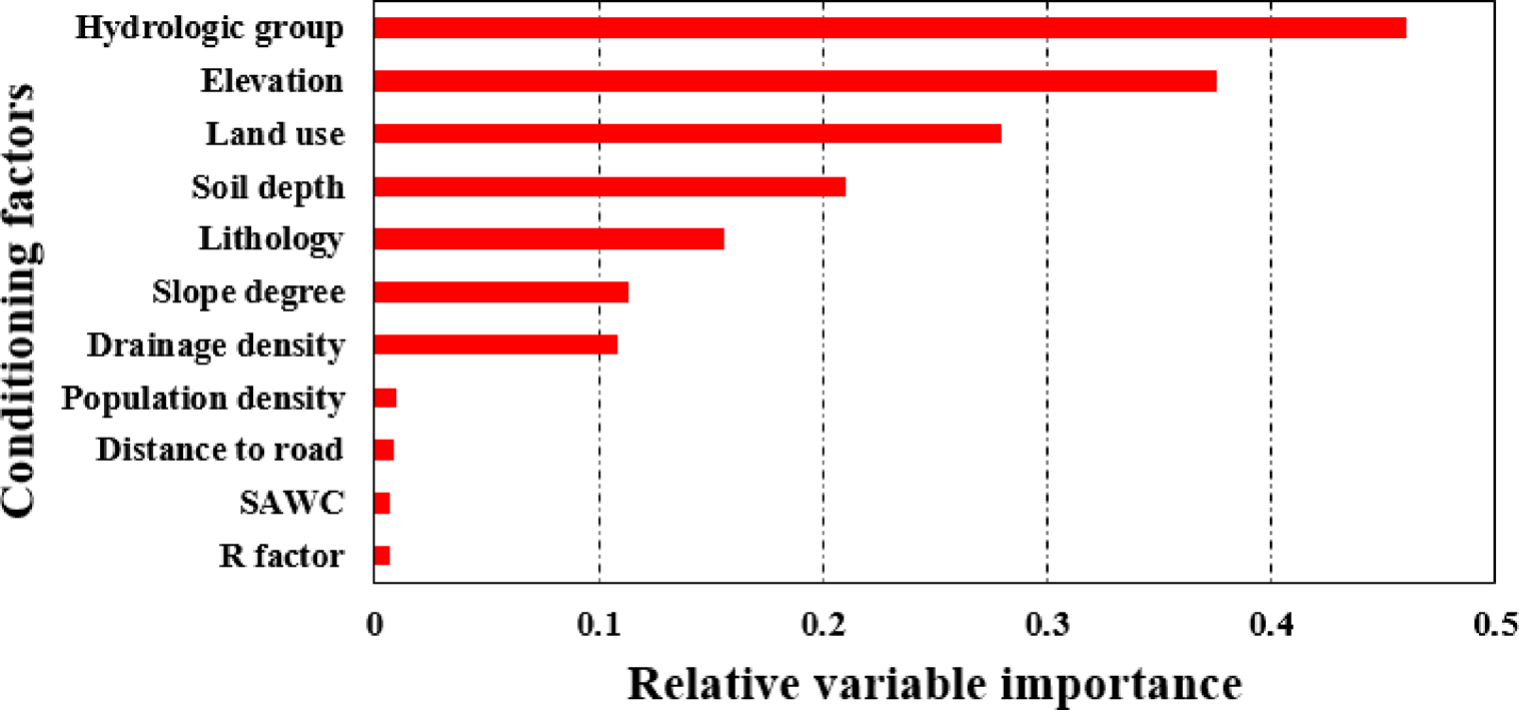
The relative importance of explanatory variables using the Jackknife test.

We conducted a jack-knife test to evaluate the relative importance of each variable included in our soil erosion hazard mapping^68^. Sensitivity analysis examines how variations in input parameters affect a model’s output, thereby identifying which inputs the model is most responsive to^69^. This sensitivity analysis method entails running the model with all explanatory factors except one, then repeating the process by eliminating each factor individually. The outcomes of the jack-knife test for the variables examined in this study are illustrated in Fig. 8. Each horizontal red bar in the figure represents the model’s accuracy, measured by AUC, derived from eliminating the corresponding variable showed on the left side of the graph. HSG, elevation, land use, and soil depth proved to be the most important soil erosion factors in this study (Fig. 8). In contrast, R, soil available water content (SAWC), distance to road, and population density had the lowest impact on erosion.

### Soil erosion susceptibility models

**Fig. 9:**
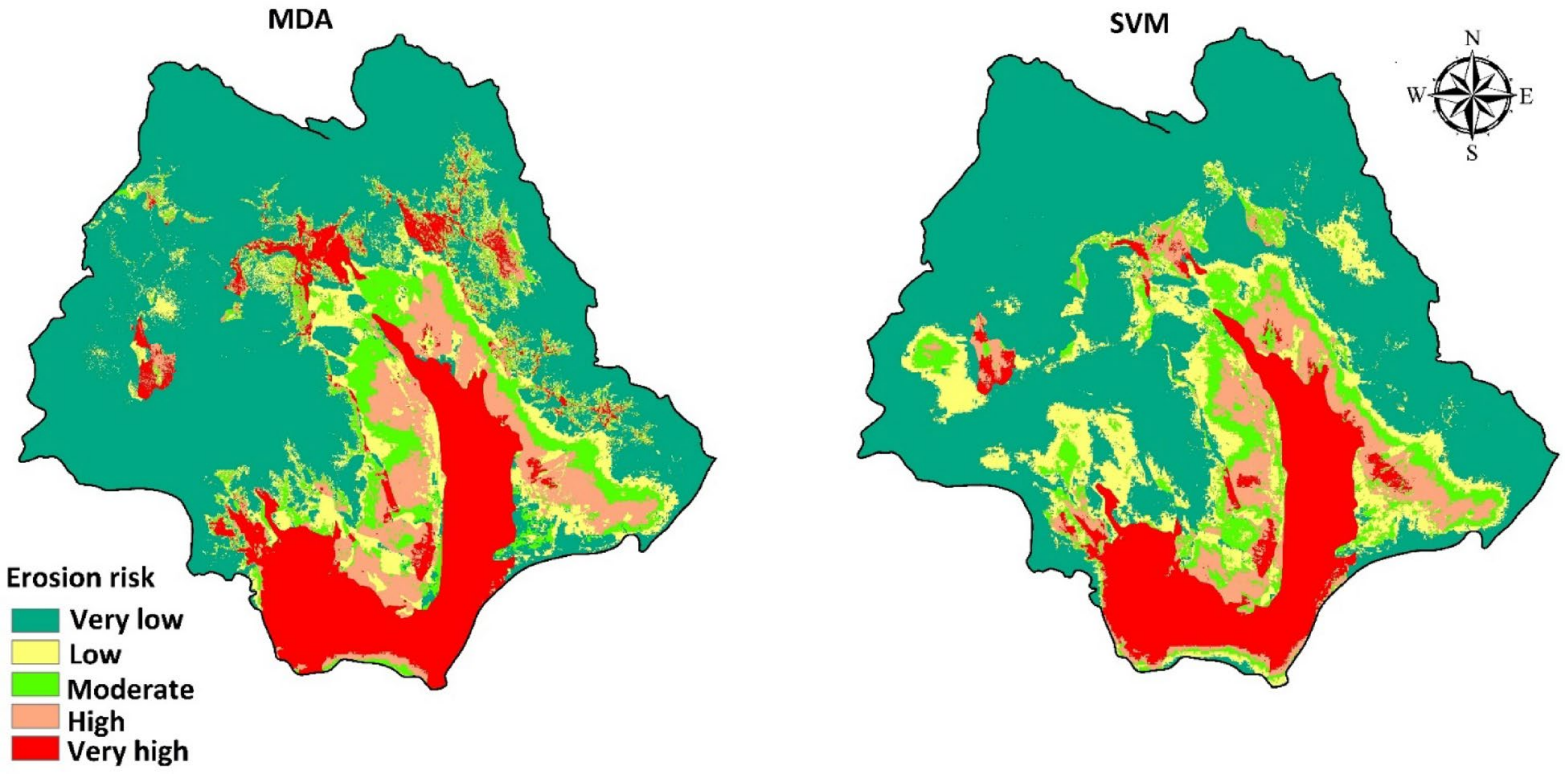
Soil water erosion risk map for MDA and SVM models using ArcGIS software (version 10.8, https://www.esri.com).

We used the natural break method in ArcMap to analyze and map the susceptibility of soil erosion in the Halil River watershed derived from the predictions of the machine learning models. The percentage of the area of the watershed occupied by each of the five classes ranging from very low to very high was computed for the predictions made by each of the two machine learning models (Fig. 9). The soil erosion maps obtained by SVM and MDA were prepared with resolution 30 m. The findings show some differences in the spatial distribution of the classes generated by each model, although the highest potential for soil erosion clearly is in the southern and central parts of the watershed, which are agricultural lands, in some places located at this part of the study area, low susceptible areas exist.

The SVM model predicts that the total area of low, moderate, and high soil erosion susceptibility classes is 14.1% of the area of the watershed (Table 5). The largest area (71.5%) of the watershed (6583.2 km^2^) is within the very low soil erosion susceptibility class. The very high erosion class, however, covers 14.2% of the watershed. In MDA, the total area of low, moderate, and high soil erosion susceptibility classes is 21.21% of the area of the watershed. The largest area (61.5%) of the watershed (5662.2 km^2^) is within the very low soil erosion susceptibility class. The importance of this area for agriculture highlights the need to prioritize it for management measures to mitigate the erosion risk.

**Table 5:** Percentage area of susceptibility classes in MDA and SVM models.

	SVM		MDA	
	Area (km^2^)	Area (%)	Area (km^2^)	Area (%)
Very low	6583.2	71.5	5662.2	61.5
Low	681.3	7.4	592.1	6.4
Moderate	294.4	3.2	628.8	6.8
High	330.1	3.5	737.2	8.01
Very high	1314.4	14.2	1583.2	17.2
Total	9203.5	100	9203.5	100

Similarly, the soil erosion susceptibility map derived using the MDA model shows that the largest percentage of the watershed (61.6%) is located in the very low soil erosion susceptibility. However, 17.2% of the watershed is within the very high erosion class.

### Model validation and accuracy

**Fig. 10:**
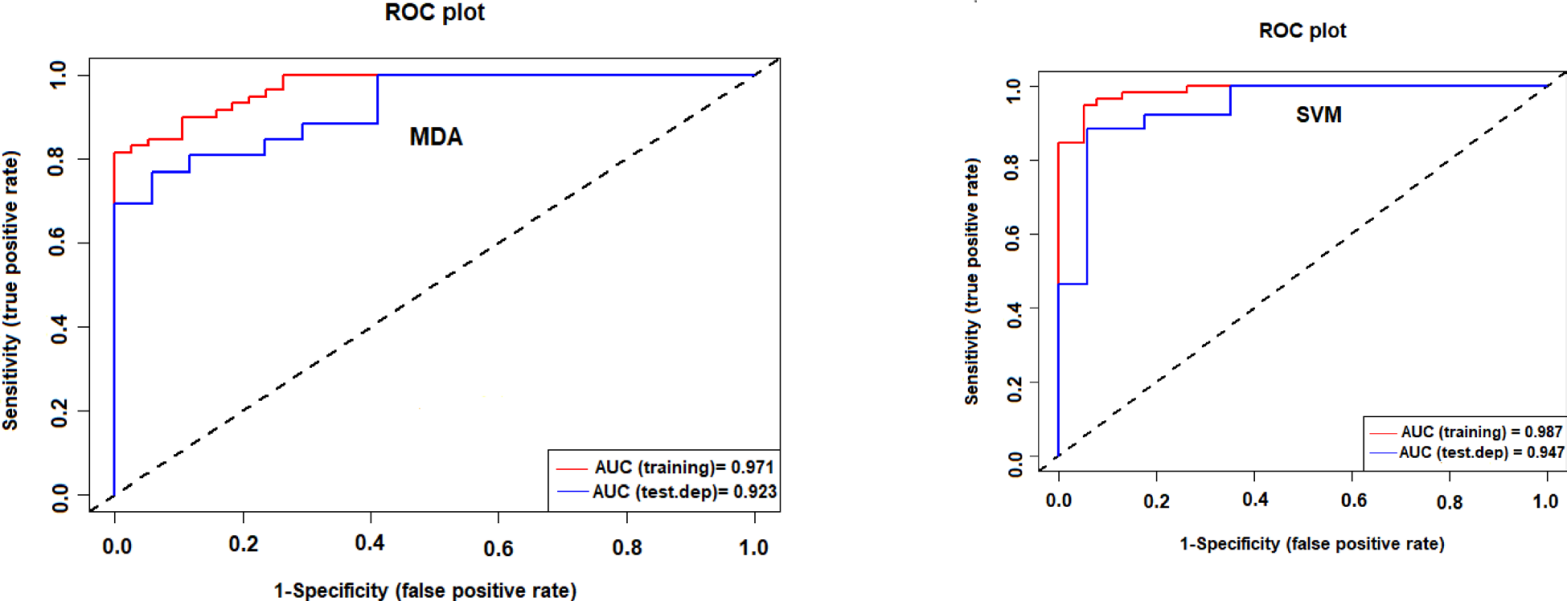
Performance evaluation of the MDA and SVM models by ROC and AUC curves.

The accuracy of the SVM and MDA models was assessed using the ROC curve, precision, the popular recall (sensitivity), TSS, F_Score, Overall Accuracy metrics. The results show that the SVM model (AUC = 94%, TSS = 0.85, Recall = 0.93, Precision = 0.91, F_score = 0.87, Accuracy = 0.90) performs a little better than the MDA model (AUC = 92.3%, TSS = 0.81 Recall = 0.88, Precision = 0.89, F_score = 0.84, Accuracy = 0.87) model (Fig. 10; Table 6). We thus selected the map created by SVM for subsequent calculations and analysis presented in the next section.

**Table 6:** Accuracy assessment of the fitted models on soil erosion data.

Metrics	SVM	MDA
AUC	0.94	0.923
TSS	0.85	0.81
Recall	0.93	0.88
Precision	0.91	0.89
F_score	0.87	0.84
Accuracy	0.90	0.87

### Soil erosion and crop water productivity (CWP) impacts

Areas and yields of the main crops produced in the Halil River watershed were determined to calculate potential losses of CWP due to soil erosion. Based on the land-use map, about 73,780 ha of the watershed is used for agriculture, including orchards. Further, more of the total area of the agriculture and orchard lands lie within the moderate to very high soil erosion susceptibility classes.

We considered average provincial yields for this study because watershed-based data on yield were not available for the Halil River watershed. The average yield of wheat (1.71 ton ha^−1^ year^−1^), dates (5.25 ton ha^−1^year^−1^), citrus (13.5 ton ha^−1^year^−1^), and tomatoes (19.34 ton ha^−1^year^−1^) thus apply to Kerman Province. Assuming that erosion-prone areas of agricultural land lose 20% of the CWP in cultivating the main crops (pessimistic scenario; Table 7), the potential losses of total CWP (TCWP) of wheat, dates, citrus, and tomatoes would be 0.43, 2.47, 4.30, and 4.70 kg m^−3^, respectively. The potential green CWP is higher than the blue CWP for all cultivated crops.

In the case of the realistic scenario, 0.32, 1.86, 3.23. 3.53 kg m^−3^ of the TCWP for, respectively, wheat, dates, citrus, and tomatoes would be reduced under the influence of soil erosion. Corresponding values for the optimistic scenario are 0.22, 1.24, 2.15, and 2.35 kg m^−3^ of TCWP. In this scenario, green CWP is higher than blue CWP as well. Also, economic loss (×10^4^US$) of the main crops production under different scenarios have been shown in Table 8. The average production of wheat, dates, citrus, and tomatoes in the watershed were 119762.5, 183677.6, 462484.3 and 402,278 tons, respectively. In the case of the realistic scenario, 21,557, 140,517, 124,872 and 77,841(×10^4^) US$ for, respectively, wheat, dates, citrus, and tomatoes would be lost under the influence of soil erosion. Economic loss of wheat, dates, citrus, and tomatoes production would be 28,743, 187,353, 166,494 and 103,782 (×10^4^) US$, respectively, under pessimistic scenario. Corresponding values for the optimistic scenario are 14,371, 93,675, 83,247 and 51,893(×10^4^) US$.

**Table 7:** Blue crop water productivity (BCWP), green crop water productivity (GCWP), and total crop water productivity (TCWP) loss of the main crops under different scenarios.

CWP Crop	Pessimistic scenario			Normal scenario			Optimistic scenario		
	Blue	Green	Total	Blue	Green	Total	Blue	Green	Total
Wheat	0.14	0.29	0.43	0.11	0.21	0.32	0.07	0.14	0.22
Dates	0.05	2.42	2.47	0.04	1.82	1.86	0.03	1.21	1.24
Citrus	0.18	4.13	4.30	0.13	3.09	3.23	0.09	2.06	2.15
Tomatoes	1.12	3.58	4.70	0.84	2.69	3.53	0.56	1.79	2.35
Total	1.49	10.42	11.91	1.12	7.81	8.93	0.74	5.21	5.95

**Table 8:** Economic loss (×10^4^US$) of the main crops production under different scenarios.

Economic loss Crop	Pessimistic scenario	Normal scenario	Optimistic scenario
Wheat	28,743	21,557	14,371
Dates	187,353	140,517	93,675
Citrus	166,494	124,872	83,247
Tomatoes	103,782	77,841	51,893
Total	486,372	364,787	243,186

## Discussion

According to the results, there was no significant difference between observed and estimated sediment and the EPM model had good performance. The Erosion Potential Method (EPM) model could be applied for different slopes^56^. In some researches with arid and semi-arid climate^9,51^, especially in Iran, in conditions of similar to the study area, despite of the steep slopes, RUSLE has been applied which has high topographic sensitivity and has been developed for low slopes^52^. For example, in a similar region in Iran, the RUSLE and machine learning models were integrated in Talar Watershed^9^. This is the advantage of this study and the weakness of mentioned studies.

Evaluating the relative importance of explanatory factors showed that HSG, elevation, land use, and soil depth proved to be the most important soil erosion factors in this study. In contrast, R, soil available water content (SAWC), distance to road, and population density had the lowest impact on erosion. The high importance of HSG aligns with the findings of Faouzi et al.^70^, but is counter to the results of Khosravi et al.^18^ who found HSG to be least important of the factors used in their soil erosion study.

We divided HSG values into four categories based on its potential for runoff. Category “A” represents sites with high permeability and low runoff. In contrast, category “D” includes sites with low permeability and high rates of runoff and erosion^70^. In this study, areas categorized as the Category “C” were found to be particularly susceptible to soil erosion.

Elevation is the second most effective factor for soil erosion susceptibility, which is in compatible with findings of Mosavi et al.^48^, Band et al^71^. and Roy et al^72^.. Despite the common assumption^51^ and finding of other research^9,14^ that areas at higher elevations are more prone to erosion than low elevation sites due to their higher rainfall, steeper slopes, and thinner soils, a different trend is evident in our study area: higher elevation areas experience less soil erosion than lower elevations ones because of the high density of agricultural lands at low elevations.

Land use is a momentous anthropogenic effect on soil loss in our study area. The development of agricultural areas and unsustainable agricultural practices have increased soil loss, consistent with the results of Barakat et al.^15,^ Band et al. ^14^and Arabameri et al^73^.. Agricultural tillage enhances soil infiltration but also contributes to significant soil losses. The use of machinery and regular plowing changes the physical properties of the soil, impacting surface runoff and increasing erosion. These processes are a topic of discussion by Silva et al.^74^. In addition, the significance and underlying factors of soil erosion vary greatly between cases, as each unique situation involves distinct types of land use and land cover. Consequently, it is crucial for planners and managers to understand the capacity and potential of various land uses to effectively prevent or mitigate soil erosion. The R factor was the least important of the variables used in this study. The northern part of the watershed lies at higher elevations and receive more rainfall than other areas, thus one might expect higher rates of erosion there. However, the southern and central plains experience far more soil erosion than the northern region. These areas, unlike those in the north, are dominantly agricultural lands, in any case, the differences in precipitation between the central and northern parts of the watershed are not great.

The results of accuracy assessment of ML models showed that the SVM model performs a little better than the MDA model. This result is in agreement with results of several other recent studies^53,75,76^. We thus selected the map created by SVM for subsequent calculations and analysis presented in the next section. In previous researches of natural hazard modeling such as avalanches and rockfalls^77^, piping erosion^59^, flood^78^, groundwater potential mapping^79^ and landslide^66^ also have been determined that the SVM model had a good performance. The regions with high erosion risk were identified.

Finally, the loss of blue, green, and total crop water productivity of some main cultivated crops (wheat, dates, citrus, and tomatoes) in the study watershed was assessed under pessimistic (20%), optimistic (10%), and normal (15%) scenarios of crop water productivity loss. According to results, agricultural lands are mostly located in areas of moderate to very high erosion risk. The economic losses associated with CWP decline are particularly alarming. Under the realistic scenario, the estimated total loss reached over $364,787 (×10⁴) US$, with wheat, dates, citrus, and tomatoes showing the highest vulnerability. Such losses directly threaten rural livelihoods and national food security, especially given the reliance of Iran’s economy on agricultural products in arid and semi-arid regions. These findings strongly suggest that soil conservation measures must be integrated into agricultural and water management policies. Strategies such as conservation tillage, mulching, cover crops, and terracing can help reduce erosion while simultaneously enhancing soil moisture retention and water productivity. In addition to the economic implications, the decline in CWP itself represents a critical loss in resource efficiency. Such reductions mean that for every cubic meter of water consumed, substantially less food is produced, which in arid and semi-arid regions like Iran translates into severe water wastage. This issue is particularly important because water availability, rather than land, is the limiting factor for agricultural expansion in these regions. The differences observed across the three scenarios also demonstrate the sensitivity of CWP to soil erosion: even a 10% reduction in productivity results in substantially less crop production per unit of water consumed.

This study provides a novel framework for evaluating the consequences of soil erosion in terms of both natural resource degradation and agricultural water efficiency. Future research should combine erosion modeling with crop growth and hydrological simulations to capture the feedbacks between soil, water, and crops under changing climate conditions. Policymakers should use these insights to design site-specific erosion control measures and promote sustainable land management practices to safeguard food production and water security in vulnerable regions like the Halil River watershed.

## Conclusion

This study investigates crop water productivity (CWP) losses of some major crops in the Halil River watershed, Iran, under three soil erosion scenarios. Two machine learning algorithms (SVM and MDA) and 11 conditioning factors (elevation, lithology, land use, hydrologic soil group, soil depth, drainage density, SAWC, population density, slope, R factor, and distance to road) were used to spatial model soil erosion susceptibility. Of these factors, hydrologic soil group (HSG), elevation, and land use had the greatest impact on soil erosion susceptibility. The accuracy of the soil erosion susceptibility maps created by the two machine learning models was validated using ROC and showed that the SVM algorithm is more accurate. Comparison of soil erosion susceptibility and land-use maps showed that all agricultural areas are located in erosion-susceptible zones. The total CWP losses of the main cultivated crops (wheat, dates, citrus, and tomatoes) were estimated to be 0.22, 1.24, 2.15, and 2.35 kg m^−3^ in an optimistic scenario, respectively. For a more realistic scenario, the CWP values of these crops are 0.32, 1.86, 3.23, and 3.53 kg m^−3^, respectively. In a pessimistic scenario, the corresponding values are 0.43, 2.47, 4.30, and 4.70 kg m^−3^. Our study clearly shows that soil erosion leads to substantial CWP losses in the Halil River watershed, calling attention to the need to implement rehabilitation and conservation measures in drought-prone agricultural areas of Iran.

Taking into account the satisfactory performance of the SVM model in our study, we suggest applying this methodology to other regions with varying characteristics. This broader application would help validate the model’s generalizability and effectiveness across diverse contexts.

In addition, this research contributes a novel integration of machine learning, erosion modeling, and water productivity assessment, offering a replicable approach for analyzing erosion-induced agricultural losses. It addresses global concerns related to land degradation, food security, and sustainable water use, key challenges underlined by international frameworks such as the UN Sustainable Development Goals (SDGs 2, 6, 13, and 15). Given the growing pressures of climate change and population growth, the methodology and insights from this work can inform regional planning and global strategies aimed at protecting agricultural productivity in erosion-prone and water-scarce environments worldwide. The model’s scalability also enables its application in other vulnerable regions, contributing to a broader scientific understanding of erosion–agriculture dynamics in a changing world.

This study, while offering valuable insights into the effects of soil erosion on crop water productivity (CWP) in the Halil River watershed, is subject to some limitations.

One of the primary limitations of this study, which is common in other spatial modeling research, is the use of input variables collected at varying scales worldwide. Although all input data were resampled to a uniform spatial resolution of 30 m, the original data collection and sampling were conducted at different scales. This remains an unavoidable constraint for now.

Also, the analysis relied on statistical reports due to the lack of access to high-resolution data on crop yields. This reliance may introduce uncertainties in the calculated CWP values. In addition, the absence of spatially explicit data on the location of individual crop fields limits the ability to analyze the spatial variation of CWP across the watershed. Looking ahead, there is considerable potential for advancing this research through more comprehensive and integrated approaches. Future studies would benefit from dynamic modeling frameworks that incorporate multi-year datasets and climate change projections to assess how temporal variations in weather patterns may influence erosion severity and water productivity. Integrating crop simulation models with hydrological models could offer a more detailed understanding of the interactions between soil, water, and crop growth under different management and erosion conditions. Ground-based measurements of soil loss, crop yield, and irrigation practices will be essential for validating model assumptions and improving reliability. Also, it is also suggested that future studies in other areas employ deep learning models and compare their performance with that of basic machine learning models.

## Data Availability

The data that support the findings of this study are available on request from the corresponding author.
